# Current strategies for ligand bioconjugation to poly(acrylamide) gels for 2D cell culture: Balancing chemo-selectivity, biofunctionality, and user-friendliness

**DOI:** 10.3389/fchem.2022.1012443

**Published:** 2022-09-20

**Authors:** Alexis Wolfel, Minye Jin, Julieta I. Paez

**Affiliations:** Developmental Bioengineering, University of Twente, Enschede, Netherlands

**Keywords:** hydrogels, bioconjugation, 2D cell culture, chemical reactivity, free radical polymerization, poly(acrylamide) gel, chemo-selectivity, biochemical properties

## Abstract

Hydrogel biomaterials in combination with living cells are applied in cell biology, tissue engineering and regenerative medicine. In particular, poly(acrylamide) (PAM) hydrogels are frequently used in cell biology laboratories as soft substrates for 2D cell culture. These biomaterials present advantages such as the straightforward synthesis, regulable mechanical properties within physiological range of native soft tissues, the possibility to be biofunctionalized with ligands to support the culture of living cells, and their optical transparency that makes them compatible with microscopy methods. Due to the chemical inertness and protein repellant properties of PAM hydrogels, these materials alone do not support the adhesion of cells. Therefore, biofunctionalization of PAM gels is necessary to confer them bioactivity and to promote cell-material interactions. Herein, the current chemical strategies for the bioconjugation of ligands to PAM gels are reviewed. Different aspects of the existing bioconjugation methods such as chemo-selectivity and site-specificity of attachment, preservation of ligand’s functionality after binding, user-friendliness and cost are presented and compared. This work aims at guiding users in the choice of a strategy to biofunctionalize PAM gels with desired biochemical properties.

## 1 Introduction

Hydrogels are crosslinked polymeric networks that can uptake and retain large amounts of aqueous fluids. This high water swelling ratio, their softness, and the possibility to regulate their biophysical and biochemical properties have made hydrogels the preferred biomaterials to mimic the extracellular matrix (ECM) that supports living cells in their native microenvironment. Therefore, hydrogels with regulated properties are extensively used as supporting materials in cell biology, tissue engineering and regenerative medicine applications.

Poly(acrylamide) (PAM) hydrogels are synthetic matrices widely used as substrates for 2D cell culture. These reductionistic *in vitro* models allow to investigate how cells sense and respond to variations in this synthetic microenvironment. For instance, the effects of hydrogel’s elasticity and/or biochemical cues on cell adhesion, spreading, migration, differentiation and protein expression have been reported. (Pelham and Wang, 1997; Heras-Bautista et al., 2019) PAM hydrogels present important advantages that explain their extended use in cell biology laboratories in the last 25 years. PAM is simple to synthesize and cost-effective, its mechanical properties (e.g., elasticity) can be easily regulated within the physiological range of native soft tissues, and its optical transparency makes it compatible with microscopy methods.

**FIGURE 1:**
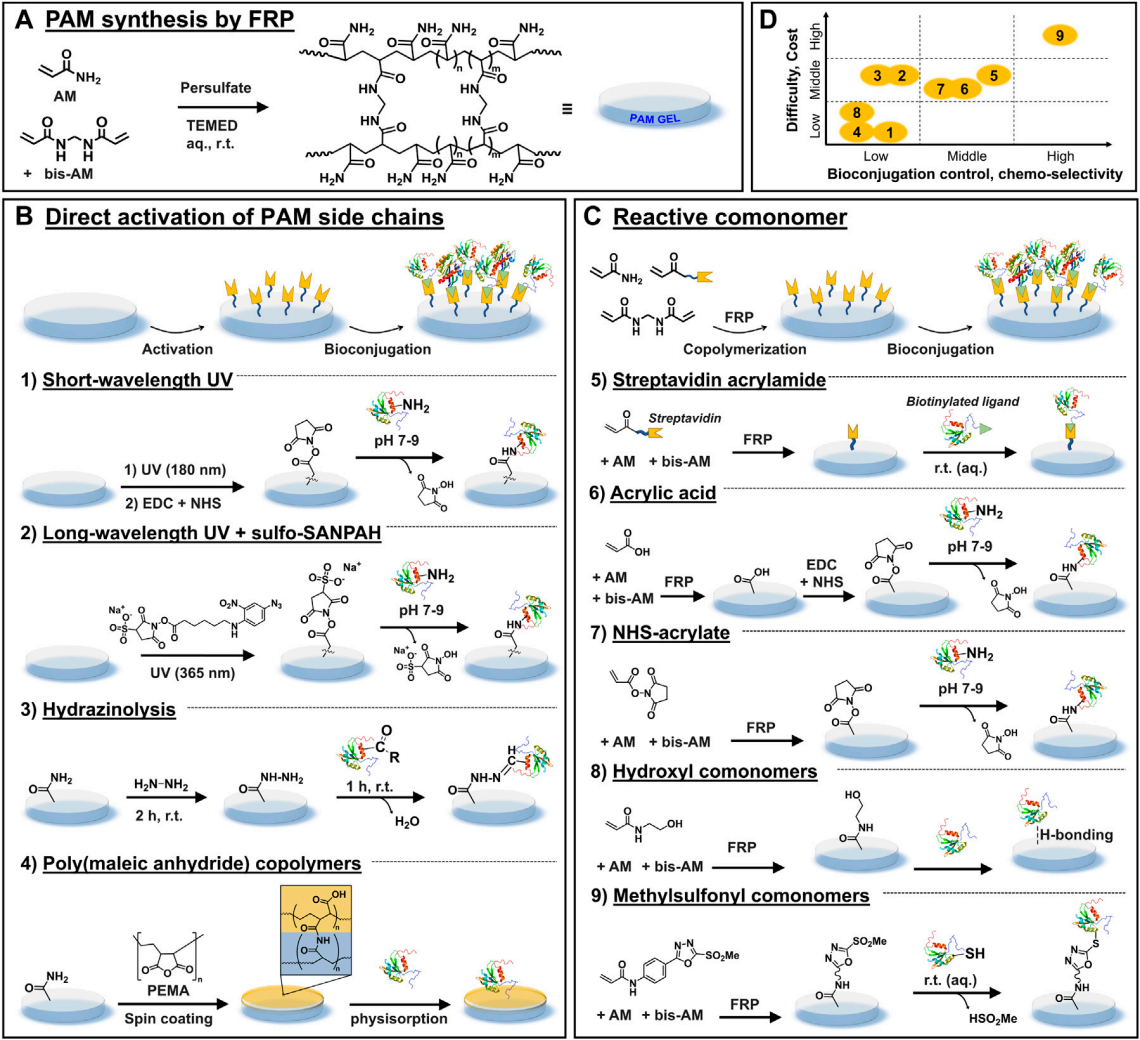
Most used chemical strategies for PAM gels biofunctionalization. **(A)** General scheme depicting the synthesis of PAM gels *via* FRP. **(B)** Chemical strategies based on the direct activation of the side chains of PAM after FRP, which is followed by ligand bioconjugation. **(C)** Chemical strategies whereby reactive comonomers are incorporated during FRP, followed by a bioconjugation step. **(D)** Overall qualitative comparison of the different chemical strategies in view of their experimental difficulty and cost vs. control of bioconjugation process and chemo-selectivity.

PAM hydrogels are typically synthesized by the acrylic-based free radical polymerization (FRP) of acrylamide (AM) and the crosslinker bis-acrylamide (bis-AM), using persulfate and TEMED as redox initiators (Figure 1A). For comfortable handling during cell culture and imaging, PAM is frequently prepared as thin film (thickness < 100 µm) and covalently attached to conventional microscopy glass coverslips, (Pelham and Wang, 1997), although attachment of thicker film gels to plastic multiwell culture plates is gaining momentum towards high-throughput studies. (Díaz-Bello et al., 2019).

For robust experimentation and reproducible outcome during culture of adherent cells on PAM gels, precise tailoring of mechanical and biochemical features of PAM substrates is desirable. Mechanically tunable PAM gels (for example, with regulable elasticity characterized by the Young’s modulus E in the range of 0.1–100 kPa) can be obtained by adjusting the bis-AM crosslinker concentration and/or the total content of acrylamide monomers in the precursor solution, (Denisin and Pruitt, 2016), and numerous standard protocols can be found in the literature. (Tse and Engler, 2010; Paez et al., 2018) In contrast, precise modulation of the biochemical properties (e.g., cell-adhesiveness to promote cell-material interactions) remains challenging because biofunctionalization of these materials is not straightforward. The reason is that PAM hydrogels are intrinsically protein repellent and chemically inert, with the lateral amide groups being poorly reactive under mild aqueous conditions. Mild conditions are required to preserve the integrity and bioactivity of bound ligands post-functionalization. Thus, various chemical strategies to enable the controlled bioconjugation of ligands to PAM gels have been developed. These strategies can be summarized in two main groups: those that target the direct “activation” of the side chains of PAM after FRP, followed by ligand bioconjugation (Figure 1B); and those based on the incorporation of reactive monomers during FRP followed by bioconjugation Figure 1C).

In this mini review, the most used chemical strategies developed for controlled biofunctionalization of PAM gels for cell culture are presented and compared. Commercial PAM hydrogels have become available; thus they are also introduced. Advantages and limitations of the diverse approaches are discussed, especially in terms of chemo-selectivity and site-specificity of binding, preservation of ligand’s functionality after conjugation, user-friendliness and cost (wherever known).^1^ The aim of this work is to guide users in the choice of a strategy to fabricate PAM gels with tailored biochemical properties for 2D cell culture.

## 2 Chemical strategies for poly(acrylamide) gels biofunctionalization

### 2.1 Direct activation of poly(acrylamide) side chains

After gel fabrication, PAM chains are (photo)chemically activated, generally under harsh conditions, to introduce reactive groups that are later used as anchoring groups for biofunctionalization. In a second step, the bioligand is covalently coupled to activated PAM gels (Figure 1B).

#### 2.1.1 Short-wavelength UV irradiation

PAM hydrogels are photochemically activated by short wavelength UV light irradiation (λ = 180 nm, 2–4 min) that forms ozone as well as peroxides and other reactive species on the PAM chains. (Tseng et al., 2011) The substrates are then incubated in EDC/NHS solution for chemical activation (in 10 mM MES buffer, pH 5.5, 15 min, r. t.), followed by incubation with biomolecules that present amine groups, such as fibronectin or fibrinogen (in 10 mM HEPES, pH 8.5, 1 h, r. t.) (Figure 1.B1). (Tseng et al., 2012) A photomask can be used to create ligand micropatterns with good spatial resolution.

This method is simple and has been reported to enable biofunctionalization of PAM hydrogels in a reproducible and homogeneous fashion. However, this procedure uses harsh photoirradiation conditions that might alter the mechanical strength of modified vs. bare PAM gels. Finally, the lack of chemo-selectivity might lead to poor control of the ligand loading and functionality.

#### 2.1.2 Long-wavelength UV irradiation and sulfo-SANPAH

This is one of the most used strategies for PAM hydrogel bioconjugation with amine-bearing ligands. It is mediated by the commercial reagent sulfo-SANPAH, a photoactivatable heterobifunctional molecule. (Pelham and Wang, 1997) The PAM gel is incubated in a solution of sulfo-SANPAH and exposed to longer-wavelength UV light (λ = 330–365 nm, r. t., at variable exposure times and intensity) (Figure 1.B2). Upon light illumination, sulfo-SANPAH forms highly reactive nitrene groups that bind nonspecifically to the PAM chains and introduce sulfo-succinimidyl groups. Typically, after two rounds of the activation step, the resulting hydrogel is immediately incubated in a solution of ligand (r.t., incubation times from 2 to 16 h). Note that the bioligand immobilization must be performed immediately after the sulfo-SANPAH step to avoid the inactivation of sulfo-succinimidyl groups *via* hydrolysis. Using this strategy, different ECM proteins such as collagen I were bound to PAM gels to mediate cell adhesion, for example, to study the traction forces generated during cell migration (Dembo and Wang, 1999) or to assess the relationship between energy expenditure during cell spreading and cellular mechanoresponse. (Xie et al., 2021)

In comparison to the short-wavelength irradiation strategy shown above, sulfo-SANPAH strategy is milder and renders higher efficiency of bioconjugation, while it keeps the simplicity of NHS ester/amine coupling. However, sulfo-SANPAH is very pricy (∼350 € per 50 mg) and has short shelf life. There is a lack of consensus on the experimental conditions for the photo-illumination step, e.g., in terms of light intensity and irradiation time. Consequently, highly variable irradiation doses can be found in the literature, which is reflected in high variability in the efficiency of bioconjugation. Additionally, poor site-selectivity of the NHS ester/amine binding has been suggested to cause poor binding efficiency and impaired ligand’s bioactivity, leading to inconsistent results in cell response. (Farrukh et al., 2016)

#### 2.1.3 Hydrazinolysis

**FIGURE 2:**
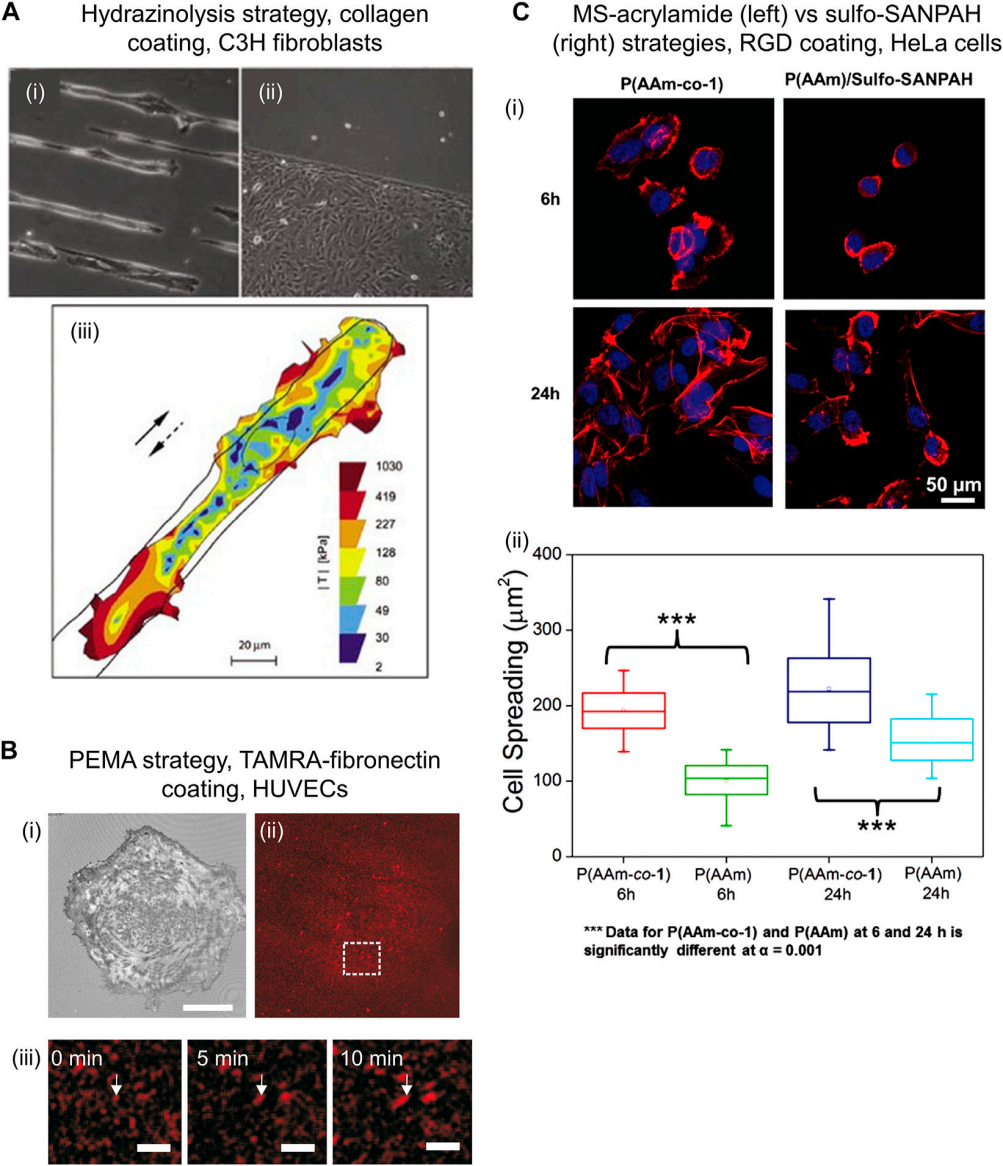
Examples of cells cultured on PAM hydrogels that were biofunctionalized through diverse chemical strategies. **(A)** Bright field images of C3H fibroblasts on PAM gels patterned with collagen I using hydrazinolysis strategy on 15 µm stripes (i) and bottom half part of the image (ii). Cells kept viable after 3 days of seeding. Traction map of C3H fibroblast while moving along a 25 µm wide stripe of collagen I (full and dashed arrows indicate original and reversed direction, respectively) (iii). Reproduced with permission from Ref. (Damljanovic et al., 2005). Copyright 2005 Future Science Ltd. **(B)** Reflection interference contrast microscopy image showing adhered HUVECs after 30 min seeding (i) on PAM gels functionalized with TAMRA-fibronectin (ii) *via* PEMA strategy. (Pompe et al., 2011) Scale bar: 30 μm. *In situ* analysis of fibronectin fibrillogenesis shows growing fibronectin fibers over time, indicated by white arrows (iii). Scale bar: 5 μm. Reprinted from Ref. (Pompe et al., 2011). Copyright 2011, with permission from Elsevier Ltd. **(C)** Confocal fluorescence images showing Hela cells after seeding for 6 and 24 h on PAM gels functionalized with RGD peptide *via* MS-acrylamide (left panel) vs. sulfo-SANPAH (right panel) strategies (i). Cell spreading area on PAM-MS gels was significantly larger than on sulfo-SANPAH gels, demonstrating effective and chemo-specific ligand immobilization (ii). Reproduced with permission from Ref. (Farrukh et al., 2016). Copyright 2016 John Wiley and Sons, Inc.

The amide groups of PAM chains are chemically converted to hydrazide groups by reaction with hydrazine (2 h, r. t.), followed by treatment of the gel with acetic acid (1 h, r. t.). The formed hydrazide groups are subsequently coupled to aldehyde or ketone groups located in biomolecules, forming hydrazone bonds. (Timofeev et al., 1996) Aldehyde or ketone bearing biomolecules can be prepared by reaction with an oxidizing agent such as sodium periodate, and then covalently bound to PAM-hydrazide gels (1 h, r. t.) (Lee et al., 2016) (Figure 1.B3). Following this procedure, various ECM proteins including collagen I, collagen IV, fibronectin and laminin were efficiently conjugated to PAM-hydrazide gels and enabled micropatterning for mechano-transduction assays (see Figure 2A). (Damljanovic et al., 2005) Furthermore, fibronectin-coated gels were used to study the influence of interfacial geometry on cancer stem cell tumorigenicity. (Lee et al., 2016)

This simple procedure has been reported to facilitate uniform, reproducible and efficient protein conjugation on PAM surface using inexpensive commercial chemicals. Additionally, the PAM-hydrazide hydrogels can be produced in big batches and are stable for 2 months. (Timofeev et al., 1996) However, some limitations can be mentioned. The oxidation of biomolecules prior to conjugation typically generates multiple aldehyde or ketone groups in the structure. Low control of site-selectivity might decrease ligand’s functionality and hamper precise control over loading. Moreover, hydrazone bonds are reversible and prone to hydrolysis under physiological conditions, therefore, biofunctionalized gels are only stable for about 2 days (Timofeev et al., 1996). This is inconvenient for longer-term cell cultures.

#### 2.1.4 Poly(maleic anhydride) copolymers

PAM hydrogels are spin-coated with a thin layer of commercially available poly (maleic anhydride) copolymers, such as poly (ethylene-*alt*-maleic anhydride) (PEMA), and incubated overnight (in PBS, pH 7.4). The anhydride groups of PEMA react chemically with amide groups of PAM hydrogel *via* covalent imide bond formation that leads to the attachment of a PEMA monolayer onto PAM. (Müller et al., 2018) Subsequently, PAM-PEMA gels are incubated in ligand solution, such as fibronectin (in PBS, pH 7.4., r. t., 1 h) and rinsed (Figure 1.B4). The PEMA layer promotes the binding of biomolecules *via* physical adsorption. (Müller et al., 2018) (Pompe et al., 2011) Through this approach, PAM gels biofunctionalized with fibronectin have been used to study the mechano-transduction mechanisms of HUVECs. (Müller et al., 2019) Similar gels were used to evaluate cellular traction force and fibronectin reorganization on adhered HUVECs. (Pompe et al., 2011) It was found that the cellular traction force correlated with the mobility of fibronectin during cell-driven fibronectin fibrillogenesis (Figure 2B).

An advantage of this approach is that a stable PEMA layer on PAM surface can be achieved using the inexpensive PEMA (∼70 € per 100 g) in a relatively simple procedure. Spin-coating ensures a thin and uniform layer but could also be a limitation in scaling up production. The lack of specificity and the instability of the ligand binding to the PEMA layer could be a disadvantage for those applications requiring fine control of ligand loading. However, reversible physical interaction between the bioligands and the PEMA layer is advantageous for mechano-transduction studies that require mobility of the adhesive ligands on top of PAM substrates. (Pompe et al., 2011)

### 2.2 Copolymerization with functionalized acrylic comonomers

Acrylic comonomers that present an additional functional group are copolymerized with AM and bis-AM to synthesize hydrogels with pendant functional groups. After polymerization, such groups are used to immobilize bioligands through covalent or non-covalent interactions (Figure 1C). In principle, by designing the right comonomer, any bioconjugation chemistry could be adapted to the synthesis of functional PAM copolymer hydrogels. Ideally, the comonomer should present decent water solubility and chemical compatibility with FRP conditions (i.e., not inhibiting PAM polymerization), be available from commercial sources or easy to synthesize, as well as cost-effective. Additionally, the introduction of this comonomer should not impair desirable PAM hydrogel properties for cell culture (i.e., transparency, controlled mechanical and swelling properties).

#### 2.2.1 Streptavidin acrylamide

Streptavidin-acrylamide (streptavidin-AM) is commercially available (∼370 € per 1 mg). After copolymerization, the PAM-streptavidin gels are functionalized with biotinylated ligands *via* non-covalent, strong supramolecular interactions between streptavidin and biotin (Figure 1.C5). Conveniently, this reaction occurs in aqueous media and at r. t. By this means, ligands such as laminin, fibronectin, and laminin-mimetic peptide IKVAV have been attached and micropatterned onto gels surface to allow cell attachment. (Hynd et al., 2007) Neural cells showed successful adhesion and a long-term viability on laminin and fibronectin-functionalized substrates.

The advantages of this approach are its simplicity, the use of well-established and user-friendly supramolecular chemistry to bind the ligands, and the compatibility with microcontact printing. Diverse biotinylated ligands can be attached, for which there is good availability of commercial options. In addition, the formed hydrogels are highly hydrophilic and nondegradable. However, the streptavidin-AM comonomer and the biotinylated ligands are expensive, and other PAM functionalization alternatives may be more suitable when micropatterning is not needed. In addition, the (to our knowledge) lack of material characterization on PAM-streptavidin hydrogels may restrain its utilization towards further cell studies.

#### 2.2.2 Acrylic acid

Acrylic acid (AA) is commercially available and cost-effective (∼40 € per 1 L). After copolymerization, carboxylic acids of PAM-AA gels are activated with EDC/NHS to form NHS esters, followed by covalent coupling with amine-ligands (Figure 1.C6). The bioconjugation step carries out under mild reaction conditions (1 h, r. t., near-neutral pH) in high yields and the formed amide bond is very stable. Since many biomolecules have free primary amine groups, this strategy offers wide versatility in the type of ligands to immobilize. For example, RGD peptide (Farrukh et al., 2017a), poly-D-lysine (Farrukh et al., 2017b), and laminin mimetic IKVAV peptides (Farrukh et al., 2018) have been successfully bioconjugated to PAM-AA hydrogels. Commercial availability, excellent water solubility and low cost are the most important advantages of this strategy, while potential limitations are the poor site-selectivity of coupling and alteration of various gel’s properties, as explained below.

First, the site-selectivity of the NHS ester/amine cannot be controlled, thus any amine group that is sufficiently exposed in the ligand might bind. Many biomolecules have multiple amine groups, and some can play a structural role in their bioactivity. Therefore, their immobilization through NHS ester/amine coupling can lead to impaired functionality when the amine group locates in the active sequence, such as Lys in IKVAV (Farrukh et al., 2018). A possibility to ameliorate this limitation is to add more conjugation steps mediated by biotin/streptavidin supramolecular interactions. For example, after PAM-AA synthesis and functionalization with biotin-PEG-amine *via* NHS ester/amine coupling, the biotin groups can mediate supramolecular binding of streptavidin followed by attachment of site-controlled biotinylated ligands (e.g., produced recombinantly). Using this strategy, hydrogels micropatterned with biotinylated anti-CD3 antibodies by microcontact printing, at tunable ligand density and variable gel stiffness were used to study the interplay of receptors and forces in T cell activation. (Zhang et al., 2021) Although it involves more coupling steps and the use of biotinylated ligands that need to be specially synthesized, this strategy can increase the site-specificity control of the bound molecule.

Second, the carboxylic groups in PAM-AA gels are negatively charged under physiological conditions. This effect increases the water swelling ratio that in turn can significantly modify several hydrogel’s properties such as stiffness, zeta-potential, and pore size; consequently impacting cell behavior. For example, 18 mol% content of AA duplicates the swelling capacity of the gels in comparison to bare PAM, which increases the stress relaxation of the material and affects adhesion and differentiation of cultured hMSCs. (Prouvé et al., 2021) A possible way to control excessive swelling is the use of longer-chain carboxylic acid-functionalized comonomers. (Heras-Bautista et al., 2019; Norris et al., 2021)

#### 2.2.3 NHS-acrylate

Acrylic acid *N*-hydroxysuccinimide ester (NHS-AE) (∼40 € per 1 g) is commercially available and used to prepared PAM-NHS gels for immobilization of amine-ligands. In relation to PAM-AA gels, PAM-NHS gels skip the AA activation step and can be directly used for NHS ester/amine bioconjugation (Figure 1.C7). On the flipside, NHS-AE presents low water solubility and also inhibits PAM polymerization at concentration >16 mol %, which affects the mechanical strength of resulting gels. (Kumai et al., 2021) Due to solubility issues, NHS-AE is typically dissolved in toluene and incorporated to the AM and bis-AM aqueous mixture before polymerization. Different reported protocols have either localized NHS groups at the surface (Schnaar et al., 1978) or promoted homogeneous incorporation of the monomer along the PAM-NHS gel. (Kumai et al., 2021) Presumably, a differentiated localization of NHS monomers along the gel alters its properties, as well as functionalization efficiency and cell-behavior. MCF7 and MCF10A epithelial cells were seeded on PAM-NHS gels coated with collagen-I and compared with PAM gels functionalized *via* sulfo-SANPAH strategy. No noticeable differences in cell adhesion or morphology were observed between both gels. (Kumai et al., 2021)

Despite the relative simplicity of this approach, NHS groups can hydrolyze over time, thus the ligands must be bioconjugated immediately after copolymerization. Furthermore, the hydrolysis of NHS groups results in charged carboxylic groups that can change hydrogel properties as explained above.

#### 2.2.4 Hydroxyethyl acrylate

Hydroxylated comonomers endow PAM gels with -OH moieties that are used to immobilize ligands by hydrogen bonding interactions (Figure 1.C8). Diverse ligands can be attached since most biomolecules can participate in hydrogen bonding. However, the immobilization is not chemo-selective and generally is chemically weak, offering little control over ligand loading and stability. Note that hydrogen bonding interactions can be unstable to washing steps, or to changes in temperature or ionic strength. These aspects might be important for studies that require control over ligand density.

Furthermore, hydroxyethyl acrylate (HEA) is inexpensive (∼60 € per 1 L). PAM-HEA hydrogels have been modified with fibronectin and laminin (Grevesse et al., 2014) and used to study how substrate area confinement affects collective migration of cells (Mohammed et al., 2019).

#### 2.2.5 Methylsulfonyl acrylamides

Comonomers bearing a methylsulfonyl (MS) group enable the chemo-specific covalent coupling of thiol-containing bioligands under physiological conditions (1 h, PBS, r. t.) (Figure 1.C9). In general, bioconjugating ligands through thiols renders higher site-selectivity compared to amine coupling since free thiols are much less naturally abundant in biomolecules. MS monomers were copolymerized at 2 mol% with AM and bis-AM by usual protocols. The swelling, porosity, and low protein adsorption of PAM-MS gels were comparable to that of PAM gels. (Farrukh et al., 2016) Biofunctionalization with thiol-containing bioligands was quantitative, reproducible and stable; and retention of ligand’s bioactivity allowed specific recognition by cells.

The performance of PAM-MS gels for bioconjugation has been compared with that of sulfo-SANPAH gels. (Farrukh et al., 2016) When both substrates were incubated in a bioligand solution of equal concentration, the immobilized ligand density on PAM-MS gels was three times higher compared to sulfo-SANPAH gels. Furthermore, PAM-MS gels only reacted with thiol-containing ligands while sulfo-SANPAH gels reacted with amino and thiol containing ligands demonstrating lower chemo-selectivity of the latter. Compared with PAM-MS gels, sulfo-SANPAH gels showed decreased concentration of bound RGD peptide, which impacted cell behavior by slowing down spreading kinetics and by decreasing final spreading area (Figure 2C). This evidences that selective ligand coupling is an important factor to achieve conclusive studies over cell-materials interactions.

Selective dual functionalization of PAM gels has been reported combining methylsulfonyl/thiol coupling with NHS ester/amine coupling (Farrukh et al., 2017a; Paez et al., 2018). PAM gels were copolymerized with AA and MS comonomers. PAM-AA-MS gels enabled the orthogonal immobilization of polylysine and IKVAV peptide. Neuronal cells cultured on gels *via* sequential bifunctionalization exhibited higher maturation at comparable culture times than when both ligands were incubated simultaneously (Farrukh et al., 2017a).

These reports have shown that functionalization of PAM gels with chemo-selective and quantitative coupling chemistries can impact retention of ligand biofunctionality and cell behavior. However, MS acrylamides are not commercially available, and their synthesis requires 4-5 steps, which reduces the user-friendliness of this approach for biology laboratories without synthetic capability.

### 2.3 Commercial poly(acrylamide) hydrogels

Commercial PAM hydrogels have become available. PAM gels with different biochemical and biophysical properties and in diverse culture plate configurations are commercialized under the brand Softwell® by Matrigen. (Matrigen, 2022) Collagen-modified and “easy coat” gels with controlled stiffness from E = 0.1–100 kPa are available. “Easy coat” gels contain quinone groups that can be used for covalent biofunctionalization by reacting with amine, thiols or other strong nucleophiles present in a ligand. Therefore, this approach presents poor chemo-selectivity and low control on site-specificity of binding.

The advantage of this strategy is the fast and user-friendly functionalization of the gels with biomolecules; thus, these substrates might be convenient for certain cell studies. However, as frequently occurring with commercial systems, the exact chemical composition of the gels, ligand density, or the chemistry applied for ligand immobilization (e.g., collagen) are undisclosed. The lack of information in composition, poor control over bioligand density and unsure retention of ligand’s functionality may lead to inconclusive results on cell culture results. Another limitation is the high cost of these substrates (typically >50 € per culture plate).

## 3 Discussion and perspectives

In this mini review, diverse strategies for biofunctionalization of PAM hydrogels were introduced in the context of these biomaterials’ usage for 2D cell culture. New users should select a convenient strategy after consideration of the advantages and disadvantages of each strategy, the existing capabilities in the laboratory and, most importantly, specific user’s needs in view of the biological question under investigation. The showcased methodologies present various levels of complexity, costs and requirement for especial equipment. Additionally, these strategies enable different degree of control over the loading density on the gels, as well as over stability and preserved bioactivity of the attached ligand. An overall qualitative comparison is presented in Figure 1D.

Two main approaches were distinguished: those based on the direct activation of the side chains of PAM after FRP, and those where reactive comonomers are incorporated during FRP. In general, PAM direct activation strategies are based on the use of commercial reagents, use relatively simple (although often harsh) modification conditions without the need of highly specialized equipment (being thus more user-friendly). Unfortunately, they often offer limited control on the ligand loading, lower preservation of bioactivity and variable stability of the coating over time.

Within strategies mediated by introduction of comonomers during FRP, the use of AA and MS-acrylamide comonomers are generally more chemically controlled, the latter being more chemo-specific and site-selective, ensuring good control of ligand loading and good preservation of ligand’s activity. However, MS-acrylamide strategy is more labour intensive and prohibitive for cell culture laboratories without organic synthesis infrastructure and expertise. Interestingly, we noticed that copolymerization strategies often do not report how the mechanical properties of the resulting functional PAM gels compare to pristine PAM gels. This could be a point of attention for future developments, to demonstrate to which extent a new methodology offers the possibility to independently tune mechanical from biochemical properties.

Although out of the scope of this article, specific needs such as patterning of PAM or high throughput handling could be other aspects to consider. Several methodologies allow ligand patterning using various chemical strategies as shown in previous sections. (Daliri et al., 2021) Noteworthy, the introduction of photolabile comonomers into PAM has recently been exploited to dynamically and locally tune the mechanical (Norris et al., 2021) and cell-adhesive (Missirlis et al., 2022) properties of light-responsive PAM gels. In contrast, the realization of truly high throughput PAM systems is still in its infancy. Commercially available PAM gels might provide an answer to this need in the long term; unfortunately, they are currently limited in their ligand options and are expensive. It is expected that more synthetic and fabrication efforts in the future will result in more robust, reproducible and high throughput solutions for biofunctionalized PAM hydrogels.

## Footnotes

1 The listed reagents' prices correspond to the cheapest alternative that is offered by an European provider, to the best of the author’s knowledge at the time of writing.

## Abbreviations

AA, Acrylic acid; AM, acrylamide; bis-AA, *N,N′*-methylenebisacrylamide; ECM, extracellular matrix; EDC, 1-ethyl-3-(3-dimethylaminopropyl)carbodiimide; FRP, free radical polymerization; HEA, hydroxyethyl acrylate; hMSCs, human mesenchymal stem cells; HUVECs, human umbilical vein endothelial cells; Ile-Lys-Val-Ala-Val (IKVAV), laminin-mimetic peptide; MS, methylsulfonyl; NHS, *N*-hydroxysuccinimide; NHS-AE, acrylic acid N-hydroxysuccinimide ester; PAM, poly(acrylamide); PBS, phosphate-buffered saline; PEMA, poly(ethylene-*alt*-maleic anhydride); Arg-Gly-Asp (RGD), cell-adhesive peptide; r.t., room temperature; *N*-hydroxy-sulfosuccinimide, (sulfo-NHS); sulfosuccinimidyl 6-(4'-azido-2'-nitrophenylamino)hexanoate (sulfo-SANPAH), *N,N,N′,N′*-tetramethylethane-1,2-diamine (TEMED).
